# Medical Reasons for Divorce

**Published:** 1910-11-26

**Authors:** 


### Medical Reasons for Divorce.

Some medical witnesses have appeared recently before the Royal Commission on Divorce, but there is a member of the profession whom we have no expectation of seeing under examination. If only in view of his German reputation as an authority on sex, Mr. Havelock Ellis should have been called. We have said his German reputation; for indeed of his latest book,* which appears almost simultaneously in America and Germany, no English edition exists. It is the concluding volume of a series, and from the postscript we learn the fact, told of in words of dignified, quiet resentment, that after the appearance in this country of the first volume a Government prosecution put an end to the sale of the work. Although British officialdom and British public opinion have many good qualities, the lack of prudishness is not one of them. In many instances a wrong verdict on their part results in no great harm, but when the pursuit of knowledge comes to be interfered with things are different. Just now in the political world the intense industry of Germans in this respect is being held up to admiration: scholars and scientific men of every kind have long known of it in their various departments. To take a cognate example, there are at any rate two German scientific periodicals devoted solely to the advance of knowledge on sexual matters. It seems unfortunate that from his own country, where are none too many such, a student of rare industry and power of expression should be, as it were, banished.

The author's views on medical reasons for divorce form, of course, but a small part of his treatment of the subject of divorce itself. The German Code of 1900, as Dr. Mott stated in his evidence, grants divorce for insanity, and presumably this can be obtained also in Russia and in Switzerland, since these countries permit divorce respectively by mutual consent and for "circumstances which seriously affect the maintenance of the conjugal tie." Monaco expressly grants it for such reasons as alcoholism, syphilis, and epilepsy. In Norway and Roumania too the law is liberal, as is certainly also the case in some of the United States and also in Transylvania. In New Zealand habitual drunkenness is a valid ground. A fairly common medical opinion is represented by the view of Dr. Woods Hutchinson, who argues that when there is epilepsy, insanity, moral perversion, habitual drunkenness, or criminal conduct of any kind, divorce is for the sake of the next generation not only permissible but imperative. This is, of course, the eugenic argument, and it is not so hard on the individual as might appear to a layman, because in insanity at any rate childbirth is a common cause of relapse. It is a little startling to find Mr. Gladstone not altogether opposed. "We have," he said, "many causes more fatal [than adultery] to the great obligation of marriage, as disease, idiocy, crime involving punishment for life." The author's own view includes the less in the greater, for he sums up by remarking: "There seems little doubt that the modern movement for divorce must inevitably tend to reach the goal of separation by the will of both parties, or, under proper conditions and restrictions, by the will of one party." This, however, is going beyond our subject.

* *Studies in the Psychology of Sex.* Vol. VI. *Sex in Relation to Society.* By Havelock Ellis. Philadelphia: F. A. Davis Company. 1910.

